# Development and Evaluation of a Swedish Short Version of the Questionnaire About the Process of Recovery (QPR)

**DOI:** 10.1007/s10597-019-00494-6

**Published:** 2019-10-28

**Authors:** Eklund Mona, Neil Sandra, Argentzell Elisabeth

**Affiliations:** 1 Department of Health Sciences, Mental Health, Activity and Participation (MAP), Lund University, Box 157, SE 221 00 Lund, Sweden; 2 Department of Clinical Psychology, Cromwell House Community Mental Health Team, Greater Manchester Mental Health NHS Foundation Trust, Prestwich, UK

**Keywords:** Assessment, Psychometrics, Mental illness, Recovery, Validity

## Abstract

The aim was to develop a short version of the Swedish Process of Recovery Questionnaire (QPR-Swe) for use with people with severe mental illness and to investigate its internal consistency, construct validity, known-groups validity and any floor or ceiling effects. Two independent samples were used, the first (N = 226) to develop the short version and the second (N = 266) to test its psychometric properties. A seven-item version was developed by selecting items based on item-total correlations. The QPR-Swe-7 showed good internal consistency reliability (α = 0.82). It showed moderate correlations with indicators of convergent validity (self-rated health, self-mastery and quality of life) and weak with those selected to test discriminant validity (psychiatric symptoms and level of functioning). QPR-Swe-7 differentiated between people receiving two different levels of housing support. No floor or ceiling effects were found. The QPR-Swe-7 had appropriate psychometric properties for use with people with a variety of mental disorders when a brief scale is warranted.

## Introduction

Psychiatric care has for decades been defined in accordance with a medical paradigm, and the main priority has been curing symptoms for people diagnosed with mental illness (Slade et al. 2014). In contrast, the rapidly growing recovery movement recognizes that people with experience of mental illness can live productive lives with symptoms and that many can recover (Davidson 2016). The concept of personal recovery has been defined as “a deeply personal, unique process of changing one’s attitudes, values, feelings, goals, skills and/or roles. It is a way of living a satisfying, contributing life even within the limitation caused by illness” (Anthony 1993, p. 527). Personal recovery as a concept has been underpinned by an extensive body of research (Slade et al. 2012), and various frameworks have been constructed to further conceptualize the meaning of personal recovery (Shanks et al. 2013). The concept of personal recovery together with a growing body of research has also become a guiding principle in mental health policies. One framework for recovery is the CHIME, which stands for Connectedness, Hope and optimism, Identity, Meaning and Empowerment. The CHIME framework was developed by Leamy et al. (2011) and is now used in both research and clinical settings.

It is desirable and important to know how service users assess their recovery. This can be for clinical reasons, such as when evaluating the mental health services provided for individuals and groups of service users, or research purposes. A number of instruments have been developed for these purposes, and in a review by Shanks et al. (2013) the Questionnaire about the Process of Recovery (QPR) was identified as a valuable measure for recovery. The QPR (Neil et al. 2009) was originally developed in the UK through collaboration between researchers and clinicians based on identified themes of recovery from service users (Pitt et al. 2007). Shanks et al. (2013) found the QPR to be particularly useful, because it showed very good psychometric properties and due to it covering central aspects of recovery as synthesized in the conceptual CHIME framework, based on descriptions and models of personal recovery (Leamy et al. 2011). The psychometric properties of the QPR were further examined and a shorter version proposed (Law et al. 2014). A Swedish version of the QPR (QPR-Swe) has recently been developed (Argentzell et al. 2017) and was found to have psychometric properties on par with the English version, including good internal consistency, construct validity and sensitivity to change.

People who experience severe mental illness over a long period of time may develop a psychiatric disability, defined as a lasting incapacity to manage everyday life due to mental illness (Swedish Government Official Reports 2006). Longer questionnaires can in some situations be time-consuming for people to complete and could even lead to them feeling more unwell. It is thus important to develop and use brief instruments that are easy to understand and complete given that it is imperative to gain knowledge of their personal recovery (Mausbach et al. 2009).

The proposed QPR-Swe has 16 items, but it is anticipated that a shorter version might be quicker and easier to complete and thus be potentially less challenging for the individual. This is especially important if the recovery measure is only one in a battery of routine outcome measures, which clients are being increasingly asked to complete, and where measures are completed repeatedly (Mausbach et al. 2009).

The current study aimed to develop a short version of the QPR-Swe and investigate its internal consistency, construct validity, known-groups validity and any floor or ceiling effects.

## Methods

The development and testing of a short version of the QPR occurred in two steps, using two different samples. The first sample was used to develop a pilot version. To strengthen the study design the pilot version was then tested for psychometric properties by using a second sample.

### Participants

Sample one consisted of people with mental illness (n = 226) participating in an RCT evaluating an activity-based lifestyle intervention aimed at improving activity balance and well-being. All participants gave written informed consent and the study was approved by the Regional Ethical Review Board. The participants’ mean age was 41 years and 73% were women. Self-reported mental disorders included; anxiety/depressive disorders (44%), followed by psychoses (23%) and neuropsychiatric disorders (17%). This sample is described in more detail elsewhere (Eklund and Argentzell 2016). The participants completed measures of personal recovery, and subjective perceptions of everyday activities and well-being. Their baseline scores on measures of personal recovery were used to develop a pilot version of a short-form QPR-Swe (see below).

Sample two included people with psychiatric disabilities who received housing support and participated in a cross-sectional study engagement in meaningful and satisfying activities in one’s home and the nearby surroundings. The participants gave written informed consent and the Regional Ethical Review Board approved the study. The participants’ mean age was 47 years and 51% were women. The most common self-reported mental disorders were psychoses (48%). Anxiety/depression was reported by 20% and a neuropsychiatric disorder by 22%. Sample two included two sub-samples, one living in congregate supported housing (SH) with support available 12–24 h per day, and the other living in ordinary housing (OHS) with considerably less intense outreach support, typically 1–2 h per week. The sub-samples differed on diagnoses, psychoses being more common and anxiety/depression less common in the SH group (p < 0.001).

### The Original Questionnaire About the Process of Recovery (QPR) and QPR-Swe

The original QPR (Neil et al. 2009) is a 22-item questionnaire with two subscales; (1) intrapersonal tasks involved in recovery and (2) interpersonal factors that facilitate recovery. Seventeen items address the intrapersonal subscale, which includes questions related to personal responsibilities and tasks, such as “I can take charge of my life” and “I can actively engage with life”. The interpersonal subscale consists of five items that address thoughts on how recovery may be strengthened through interpersonal relationships, for example; “Meeting people who have had similar experiences makes me feel better”. A five-point response scale is used (0 = disagree strongly, 1 = disagree, 2 = neither agree nor disagree, 3 = agree, 4 = agree strongly). The QPR has shown good construct validity in relation to well-being and quality of life and good test–retest reliability (Neil et al. 2009), but a 15-item version based only on items from the first subscale has been found to possess more robust psychometric properties (Law et al. 2014). The QPR-Swe (Argentzell et al. 2017) was developed from the original QPR 22-item version, starting with exploratory factor analysis. The Swedish authors found, as Law and colleagues had done, a version based only on subscale one to have superior psychometric properties compared to the originally proposed two-factor solution. The findings indicated excellent internal consistency (alpha = 0.92), convergent validity in relation to self-esteem and quality of life, and reasonable sensitivity to change (Argentzell et al. 2017).

### Development of the Pilot QPR-Swe-7 (Sample one)

The 16-item QPR-Swe was the starting point and the pilot short version was created by successively deleting items. The aim was to reduce as many items as possible while still covering a variety of facets of personal recovery and obtaining an alpha value exceeding 0.80. Using Sample one, the following steps were taken:

1. We deleted all items with corrected item-total correlations (CITC) of < 0.50. This is a strong requirement, since CITC > 0.30 is regarded acceptable (Streiner and Norman 2008). The higher level was chosen in order to accomplish a veritable reduction of items. This resulted in a 14 items version and alpha = 0.92.
2. We then deleted all items with CITC < 0.60 based on the 14 items version. This resulted in 9 items and alpha = 0.89.
3. These items were then discussed in the research group with the purpose of identifying possible overlapping items. Six items with unique wordings and no content overlap were identified, which also covered core areas of recovery as described by service users and researchers in the literature (Leamy et al. 2011). Finally, we reviewed the deleted items to see if any items with a unique and central content had been deleted. This led to the inclusion of one more item. The final seven items were nos. 1, 3, 5, 6, 7, 12 and 19 from the 22-item version (Neil et al. 2009). See Table 1, where digits in brackets indicate the item number in the original 22-item version. This pilot scale reached alpha = 0.86 based on Sample one and CITC varied between 0.55 and 0.69.

**Table 1:** Items in the pilot QPR-Swe-7

Items
1. I feel better about myself (1)
2. I am able to develop positive relations to others (3)
3. I am able to assert myself (5)
4. I feel that my life has a purpose (6)
5. My experiences have changed me for the better (7)
6. I can take charge of my life (12)
7. I can actively engage with life (19)

### Psychometric Testing of the Pilot QPR-Swe-7 (Sample two)

#### Data Collection

A background questionnaire was used to gather socio-demographic information and participants’ self-reported diagnoses and psychological problems. These self-reports were then converted into ICD-10 diagnoses (WHO 1993) by a psychiatrist. The validity of this procedure has been investigated in a previous study, which showed a logical pattern of associations between type of psychiatric symptoms (depressive, positive, negative and general) and diagnostic grouping (psychoses, anxiety/mood disorders and “other”) (Eklund and Sandlund 2012).

The instruments selected to assess construct validity were chosen to reflect a construct assumed to be either similar or dissimilar to personal recovery. Testing construct validity by comparing the target construct with similar ones is termed convergent validity, and comparisons with dissimilar ones reflect discriminant validity (Streiner and Norman 2008). We chose self-rated health, self-mastery and life satisfaction to investigate convergent validity and interviewer ratings of symptoms and level of functioning to test discriminant validity.

*Self*-*rated health* was assessed by the first item from the often-used SF-36 (Ware and Sherbourne 1992), which asks study participants to rate their health “overall”. The response scale has five alternatives from “Excellent” (= 1) and “Very poor” (= 5). Research has indicated that this one-item evaluation is reliable and correlates strongly with the complete SF-36 (Bjorner et al. 1996; Bowling 2005).

*Self*-*mastery* was addressed by administering the seven-item self-report scale developed by Pearlin et al. (1981) and  Pearlin and Schooler (1978). The response scale ranges from “Strongly disagree” (= 1) to “strongly agree” (= 4) and a higher score denotes a stronger sense of self-mastery. The Swedish version used in the current study has been found psychometrically sound in terms of construct validity (Eklund et al. 2012). Cronbach alpha for the current Sample two was 0.76.

In order to assess *life satisfaction*, item number one in the Manchester Short Assessment (MANSA) of quality of life (Björkman and Svensson 2005; Priebe et al. 1999) was used. It addresses general quality of life and correlates strongly with a composite quality of life index (Priebe et al. 1999). A seven-point rating scale from “Worst possible satisfaction” (= 1) to “Best possible satisfaction” (= 7) is used.

*Psychiatric symptoms* and *level of functioning* were both based on the Global Assessment of Functioning (GAF) instrument (Endicott et al. 1976). A professional assesses the severity of symptoms and social, psychological and occupational functioning for people with mental illness. The instrument has a numeric scale that ranges from 0 to 100, where a higher rating indicates less severe symptoms and a higher level of functioning, respectively. The GAF has demonstrated good inter-rater reliability after only brief training (Startup et al. 2002). All data collectors in the current study had received training regarding GAF, watching videos with fictive client cases and being calibrated against an expert GAF rater.

#### Hypotheses

It was hypothesized that QPR-Swe-7 would exhibit strong or moderate correlations with the selected indicators of convergent validity (self-rated health, self-mastery and quality of life), based on previous research showing that self-variables tend to be related with personal recovery among people with mental illness (Argentzell et al. 2017; Law et al. 2016). We hypothesized weak correlations between QPR-Swe-7 and the variables chosen to reflect discriminant validity (severity of psychiatric symptoms and level of functioning according to GAF), seen as functional recovery (Mausbach et al. 2009) and therefore theoretically different from how personal recovery is defined (Anthony 1993). The fact that the GAF ratings are performed by a professional and the QPR is a self-rating further accentuates the difference in the targeted phenomena (Streiner and Norman 2008), which has also been corroborated by a study exploring service users’ preferences regarding outcome measures (Crawford et al. 2011).

Since previous research has indicated that social integration is a vital component in personal recovery (Le Boutillier et al. 2011), we hypothesized that the QPR-Swe-7 would show known-groups validity in relation to two socially oriented variables, namely living in SH (high level of social support/interaction) versus OHS (lower level) and self-report of having seen a friend in the past week or not.

### Data Analysis

All analyses testing the psychometric properties of the pilot QPR-Swe-7 were thus based on Sample two. Cronbach’s alpha was used to calculate internal consistency reliability and CITC. Non-parametric statistics were used for further analyses since the instruments applied produced ordinal scales. Associations between variables were calculated by Spearman correlations and differences between groups by the Mann–Whitney U-test. The level for statistical significance was set at *p *< 0.05 and the software used was the SPSS version 23 (“IBM SPSS Statistics 23 core system user’s guide”).

## Results

### Internal Consistency

The internal consistency of the pilot QPR-Swe-7 based on Sample two was α = 0.82. All CITC were well above 0.30 as seen in Table 2, and no deletion of items improved the internal consistency.

**Table 2:** CITC values and alpha if item deleted for the QPR-Swe-7

	CITC	α if item deleted
Item 1	0.54	0.81
Item 2	0.53	0.81
Item 3	0.48	0.82
Item 4	0.64	0.79
Item 5	0.54	0.81
Item 6	0.60	0.80
Item 7	0.64	0.79

### Construct Validity

Convergent and discriminant validity are presented in Table 3. QPR-Swe-7 showed moderate correlations with all three indicators of convergent validity and weak with those selected to test discriminant validity.

**Table 3:** Correlations between personal recovery and the indicators of convergent and discriminant validity

Indicators	Correlation coefficient	*P* value
Convergent validity		
Self-rated health	− 0.34	< 0.001
Self-mastery	0.43	< 0.001
Life satisfaction	0.44	< 0.001
Discriminant validity		
Psychiatric symptoms	0.15	0.018
Level of functioning	0.19	0.004

### Known-Groups Validity

The QPR-Swe-7 discriminated between the SH and the OHS groups (*p *= 0.004), the SH scoring higher. Their mean (SD) was 27.4 (4.4) versus 24.9 (5.9) for the OHS group. The instrument also discerned those who had seen a friend the past week and those who had not (*p *= 0.04).

### Floor and Ceiling Effects

The lowest QPR-Swe-7 rating in Sample two was nine, which represented 0.4% of the participants. There were thus no participants who gave the lowest possible rating of seven. The highest possible score of 35 was reported by 7% of the participants. The median score was 26.5, which can be compared with the theoretically obtainable median of 21.5.

## Discussion

The QPR-Swe-7 exhibited good internal consistency reliability, and a logical set of correlations with indicator variables suggested initial concurrent and discriminant validity. The associations with the indicator variables, although somewhat weaker, followed the same pattern as for the 16-item QPR-Swe (Argentzell et al. 2017). Furthermore, the QPR-Swe-7 could discriminate between subgroups who differed with respect to access to social interaction, which is known to be of importance for attaining personal recovery (Le Boutillier et al. 2011). Preliminary known-groups validity was thus indicated.

No floor effect was detected; in fact no participant used the lowest possible rating. Limits proposed for effective measurement and the avoidance of floor or ceiling effects have been set at ≤ 5% of participants attaining the lowest possible or highest possible rating (McHorney et al. 1994). Other researchers have set > 20% as problematic (Holmes and Shea 1997). Although 7% used the highest possible rating in the current study it is thus not regarded as problematic, nor as a ceiling effect. It is also noted that that the median rating was higher than the theoretical median, which however tends to be the case with ratings of personal recovery (Fardig et al. 2011; Hultqvist et al. 2018). Furthermore, although there was no indication at all of a floor effect, there was a slight tendency towards a ceiling effect. Changing any one item for an item more likely to render a lower rating might thus strengthen the QPR-Swe-7 further.

Service users have expressed critique against some of the instruments that are commonly used in mental health services and research and request measures that take their own views into account (Crawford et al. 2011). Personal recovery would be central in this. However, people experiencing severe mental illness might sometimes become overwhelmed and tired and could find it challenging to complete long questionnaires. Developing measures that are as short as possible whilst remaining psychometrically robust is thus crucial in order to facilitate engagement in recovery-orientated practice. This brief version of QPR-Swe gives even the most severely ill an opportunity to convey their view of their own personal recovery state.

### Methodological Considerations

The QPR-Swe-7 was tested in a group of people with severe psychiatric disabilities and the sample must be regarded representative of the target group. The possibilities for an extensive data collection was limited by the participants’ disabilities, however, and only a few and often single-item measures could be administered to address construct validity. Yet, single-item measures have support among researchers (Bowling 2005; Cheung and Lucas 2014) and should not constitute a problem per se. Nevertheless, the authors acknowledge that the current study provided only a basic psychometric analysis of the QPR-Swe-7. Further studies are needed, which could explore its test–retest stability, sensitivity to change, cross-cultural validity, and a further testing of construct validity.

## Conclusion

This study has provided initial evidence that the QPR-Swe-7 has appropriate psychometric properties for use with people with a variety of mental disorders, including people with psychiatric disabilities, when a brief scale is warranted. The QPR-Swe-7 adds to the existing recovery instruments (Shanks et al. 2013) by offering a brief and easily administered scale which, for example, can be used during periods of acute distress when it might be neither appropriate nor achievable to administer longer measures. To continue with longer measures in these circumstances goes against the spirit of the recovery paradigm and the ethos of the QPR, which is underpinned by working collaboratively with service users and aimed at increasing empowerment, crucial to the concept of recovery. Further testing of the psychometric properties of the QPR-Swe-7 is however warranted.
